# Why Do These Microbes Like Me and How Could There Be a Link with Cardiovascular Risk Factors?

**DOI:** 10.3390/jcm11030599

**Published:** 2022-01-25

**Authors:** Emilia Sawicka-Śmiarowska, Anna Moniuszko-Malinowska, Karol Adam Kamiński

**Affiliations:** 1 Department of Population Medicine and Lifestyle Diseases Prevention, Medical University of Bialystok, 15-269 Bialystok, Poland; emiliasawickak@gmail.com; 2 Department of Cardiology, Medical University of Bialystok, 15-276 Bialystok, Poland; 3 Department of Infectious Diseases and Neuroinfection, Medical University of Bialystok, 15-540 Bialystok, Poland; anna.moniuszko@umb.edu.pl

**Keywords:** gut microbiome, heart, cardiovascular risk factors, cardiovascular diseases, cardiovascular system

## Abstract

Cardiovascular diseases are the most common causes of hospitalization, death, and disability in Europe. Due to high prevalence and ensuing clinical complications, they lead to very high social and economic costs. Despite the knowledge of classical cardiovascular risk factors, there is an urgent need for discovering new factors that may play a role in the development of cardiovascular diseases or potentially influence prognosis. Recently, particular attention has been drawn to the endogenous microflora of the human body, mostly those inhabiting the digestive system. It has been shown that bacteria, along with their host cells, create an interactive ecosystem of interdependencies and relationships. This interplay could influence both the metabolic homeostasis and the immune processes of the host, hence leading to cardiovascular disease development. In this review, we attempt to describe, in the context of cardiovascular risk factors, why particular microbes occur in individuals and how they might influence the host’s cardiovascular system in health and disease.

## 1. Introduction

Cardiovascular diseases lead to more than 16 million deaths worldwide annually, including 4 million in Europe [1,2,3]. It is well known that cardiovascular diseases are associated with several nonmodifiable risk factors, including age, male gender, and genetic background, as well as modifiable ones, such as lack of physical activity, improper diet, polluted environment, smoking, and other indicators of an unhealthy lifestyle [4,5,6].

It should be emphasized that despite the knowledge of classical cardiovascular risk factors, the morbidity and mortality in this group of diseases remain high [7]. Consequently, it is necessary to seek new factors that may play a role in the development of cardiovascular diseases. Emerging evidence from the literature suggests that the endogenous microflora of the human body (especially microbiota inhabiting the digestive system) might be involved in many of these crucial physiological and pathological processes [8,9,10,11,12,13]. It has been shown that bacteria form an interactive ecosystem of interdependencies and relationships with their host cells (in the majority, in the form of mutualism and commensalism) [14,15,16]. This interplay could influence both the metabolic homeostasis and the immune processes of the host, thereby becoming an important part of the pathogenic process [17].

In this review, we attempt to describe why particular microbes might occur in individuals in the context of both modifiable and nonmodifiable cardiovascular risk factors and how they could influence the host’s cardiovascular system. Furthermore, we posit that some of the effects exhibited by the above-mentioned risk factors may in fact be mediated by changes in the microbiome (Table 1 and Table 2) [18].

## 2. Association of the Microbiome with Modifiable Risk Factors

### 2.1. Overweight and Obesity

Overweight and obesity have been linked to many cardiovascular diseases, including, among others, coronary artery disease, heart failure, and stroke [57,58]. Excessive body weight might be mediated by direct effects such as structural and functional adaptations of the cardiovascular system and adipokine effects on inflammation and vascular homeostasis [58]. Indirect effects in overweight and obese individuals might be related to a higher prevalence of various diseases, such as insulin resistance, hyperglycemia, hypertension, and dyslipidemia [58].

Recently, particular attention has been paid to the role of the gut microbiome in maintaining weight. Gut microbiota were proven to play an important role in the absorption, storage, and expenditure of energy obtained from dietary intake [59]. This effect might be related to short-chain fatty acid production, the stimulation of hormones, chronic low-grade inflammation, lipoprotein, bile acid metabolism, and increased endocannabinoid receptor system activity [20].

Although the findings from animal and human studies differ, the most common result is an increase in the *Firmicutes*/*Bacteroidetes* ratio [18,60] and a reduction in microbial diversity and richness [61]. Furthermore, it was proven that *Firmicutes*/*Bacteroidetes* ratio was significantly associated with BMI, and this association continued to be significant after adjusting for confounders such as age, sex, tobacco smoking, and physical activity [19].

Ridaura et al. showed that cohousing mice harboring an obese twin’s microbiota with mice harboring a lean twin’s microbiota, due to the transmission of *Bacteroidetes* from lean mice, prevents abnormally high BMI development and obesity-associated metabolic phenotypes in obese cage mates [21]. A similar proof-of-concept study of the gut microbiome transfer effect on BMI is currently being conducted on humans [62].

A single-center, double-blind, placebo-controlled trial of two separate child cohorts who were overweight or obese revealed that the group given oligofructose-enriched inulin (probiotics) or maltodextrin as a placebo was characterized as having an increase in the genus *Bifidobacterium* and a decrease in *Bacteroides vulgatus* [22]. Despite the mentioned shifts in the gut microbiome due to probiotics supplementation [22], the results of the metanalyses published in 2015 [63] and in 2019 [64] did not confirm the thesis that pre- and probiotics had an effect on body weight or BMI. In the interventional study, in which obese individuals received a dietary intervention for over three months, after weight reduction, an increase in total bacterial abundance was revealed [23]. Furthermore, *Lactobacilli*, *Clostridium cluster IV*, *Faecalibacterium prausnitzii*, *Archaea*, and *Akkermansia* significantly increased, while *Clostridium cluster XIVa* and the *Firmicutes*/*Bacteroidetes* ratio decreased [23].

#### What Might This Mean for the Heart?

Evidence from the literature suggests that SCFA (short-chain fatty acid) production by bacteria may significantly contribute to an altered energy balance [65,66,67]. In the study of Schwiertz A. et al., total fecal SCFAs were higher in obese compared to lean subjects [67]. Additionally, the propionate concentration increased significantly from lean to obese subjects [67]. SCFA, except for succinate, seems to have a beneficial influence on many cardiovascular diseases [68]. Acetate was proven to lower both the heart rate (lowering sympathetic activity) and mean arterial pressure in vivo in radiotelemetry-implanted mice [69]. Additionally, propionate seems to participate in blood pressure regulation (reduction via GPR41 due to its influence on vascular contractibility and an increase via OLFR78 due to rennin production and release) [68,70]. This SCFA also promotes anti-inflammatory Treg cells and therefore may also reduce hypertension, atherosclerosis, and cardiac hypertrophy [71]. Propionate also participates in H3Lys23 histone propionylation, which is involved in cardiac development [68].

Oral supplementation of butyrate by nuclear factor κ-B inhibition and decreasing the pro-inflammatory state might slow atherosclerosis progression [68,72]. This SCFA also suppresses histone deacetylase to reduce myocardial remodeling in the diabetic heart [68,73].

### 2.2. Cholesterol-Rich Lipoprotein Metabolism

The role of lipid metabolism in the human body is complex, and its disturbances have important physiological implications [74]. On the one hand, lipids are the major structural components of cell membranes, energy storage, and regulation molecules [75,76,77]. On the other hand, it is well known that an abnormal lipid profile, especially a high low-density lipoprotein concentration, is one of the modifiable risk factors leading to cardiovascular diseases [6]. The gut microbiota might both modulate the amount of energy that is extracted from food during digestion and synthesize lipids and metabolites (sphingolipids, sterol, fatty acyls, and glycerolipid synthesis) that may have an impact on human health [75].

In the mice model, in which animals with the presence or absence of gut commensal were fed a diet enriched with bile acids with or without the addition of lard or palm oil, a shift in dominant gut bacterial was observed [33]. In the study, a diet enriched with lard or palm oil caused body weight gain only in mice with the presence of gut commensal [33]. The diet enriched with lard and bile acid was associated with increased microbiome richness [33]. In the study, all types of diet intervention resulted in increased proportions of *Desulfovibrionaceae* and a lack of *Erysipelotrichaceae* [33]. The relative abundance of *Lachnospiraceae* significantly decreased in the diet enriched with lard oil compared to palm oil, whereas *Ruminococcaceae* increased compared to the control group [33]. Both lard and palm oil dietary interventions resulted in a lower relative abundance of *Rikenellaceae* [33].

In the study of Fu J. et al. based on 893 subjects from the Life-Lines-DEEP population cohort, 114 OUT were associated with triglycerides, and 34 with HDL (high-density lipoproteins) [24]. The family of *Clostridiaceae*/*Lachnospiraceae* was particularly associated with LDL, and there was no correlation detected with BMI or other lipids [24]. The family of *Pasteurellaceae*, genera *Coprococcus*, and *Collinsella* species *Stercoris* were associated with triglyceride levels with a nominal significance to other lipids and had no association with BMI [24]. Furthermore, a cross-validation analysis revealed that the microbiota explain 6% of the variance in triglycerides and 4% in HDL, independent of age, sex, and genetic risk factors [24].

It was suggested that the gut microbiome might participate in cholesterol conversion to coprostanol [78]. Individuals with coprostanol-forming gut microbiota have significantly lower fecal cholesterol levels and lower serum total cholesterol [78]. Previously reported coprostanol-forming bacteria are *Eubacterium coprostanoligenes*, *Bacteroides dorei*, *Lactobacillus* sp., and *Bifidobacterium* sp. [25,26,27].

Furthermore, the gut microbiome in hypercholesterolemia patients was characterized as having a lower richness and diversity of bacterial communities compared to normocholesterolemic controls [32].

#### What Might This Mean for the Heart?

There are many ways in which the gut microbiome might play a significant role in the regulation of lipid homeostasis and plasma lipid levels and therefore affect the cardiovascular system [75,79]. To reduce cardiovascular risk due to an abnormal serum lipid profile, the gut microbiota has been targeted in dyslipidemia treatment, especially with products enhanced with *Lactobacillus* spp. [28,29,30,31,34]. A meta-analysis focusing on studies using short-chain fatty acids producing the bacteria *Lactobacillus formulations* found an improvement of LDL and total serum cholesterol but not triglycerides nor HDL [28]. Two randomized, double-blind, placebo-controlled studies showed a 4.4% total cholesterol and 6.2% LDL reduction caused by *Lactobacillus acidophilus* or *Enterococcus faecium* in subjects with a normal lipid profile and medium to moderate hypercholesterolemia [29,30]. Furthermore, *Lactobacillus reuteri* was associated with increased intraluminal bile acid deconjugation, which leads to reduced absorption of sterols other than cholesterol [34]. One study found an improvement of triglycerides but not cholesterol after short-term *Bifidobacteriae* and *Lactobacilli* coadministration in healthy subjects, whereas another study revealed a decrease in LDL, total cholesterol, and triglycerides, whereas there was an increase in HDL when using a *Bifidobacterium*/yeast extract symbiotic [31]. Furthermore, Liu et al. demonstrated that the cholesterol-lowering effect of eight weeks of rosuvastatin treatment was reflected in microbial alpha-diversity [80].

### 2.3. Tobacco Smoking

There is emerging evidence that tobacco smoking might be related to gut microbiome composition [35]. A few potential mechanisms of this phenomenon were suggested, such as oxidative stress enhancement, Th17 cell-neutrophil axis, alterations of intestinal tight junctions and intestinal mucin composition, and changes in the acid–base balance [35,81]. A review of articles published between 2000 and 2016 indicated that smoking increases the abundance of *Proteobacteria* and *Bacteroidetes* phyla (*Bacteroides* and *Prevotella* genera) and genus from *Firmicutes* phylum (*Clostridium*), with a decrease in *Actinobacteria* (genus *Bifidobacteria*) and *Firmicutes* phyla (genus *Lactococcus*) [35]. Smoking also decreased the diversity of the intestinal microbiome [35]. Interestingly, a population-based, cross-sectional study, using the Healthcare Screening Center cohort and conducted on 758 men, showed that current smokers had an increased proportion of the phylum *Bacteroidetes* with decreased *Firmicutes* and—contrary to the previously mentioned article—*Proteobacteria* compared with people who had never smoked [37]. In the study, differences between former smokers and people who had never smoked were not observed [37]. Another cross-sectional study of 249 participants selected from the Health Effects of Arsenic Longitudinal Study in Bangladesh revealed that *Erysipelotrichi*-to-*Catenibacterium* lineage (*Firmicutes* phylum) was significantly higher in current smokers compared to never smokers [36]. Furthermore, each of these taxa exhibited a dose–response relationship with packs of cigarettes smoked per day [36]. The odds ratio for the genus *Catenibacterium* comparing the mean relative abundance in current smokers with that in never smokers was 1.91 and 1.89 for the family *Erysipelotrichaceae* [36]. Furthermore, the presence of *Alphaproteobacteria* (from *Proteobacteria* phylum) was significantly greater when comparing current smokers with never smokers [36]. A dose–response association was observed for each of these bacterial taxa [36]. Interestingly, one of the studies examined not only the influence of tobacco smoking but also electronic cigarettes and revealed changes in *Bacteroidetes* phylum in the form of an increased relative abundance of *Prevotella* and decreased *Bacteroides* compared to controls [38]. This study also suggested that tobacco smoking might have a more severe effect on health than electronic cigarettes [38].

A review of prospective cohort studies that assessed the gut microbiome of 1277 infant/neonatal participants exposed to environmental smoke showed that neonates exposed to environmental smoke present a higher relative abundance of *Ruminococcus* (*Firmicutes* phylum) and *Akkermansia* [39]. Infants exposed to environmental smoke during pregnancy or postnatally have increased gut bacterial richness, particularly *Firmicutes* at 3 months of age, while 6-month-old infants born to smoking mothers had an increased abundance of *Bacteroides* and *Staphylococcus* [39]. Elevated *Firmicutes* richness at 3 months of age was associated with elevated odds of child overweight and obesity at 1 and 3 years of age [39].

#### What Might This Mean for the Heart?

The data from the literature suggest that tobacco smoke might cause dysbiosis and a decrease in gut microbiome richness [35,36,37,38,39]. Tobacco smoking was positively related to *Bacteroidetes* phylum [35,37,38,39] and some potentially pathological genera from *Firmicutes* [35,36,39] and inversely associated with beneficial genera such as *Lactococcus* [35] and *Bifidobacteria* [81]. The predominant genera in the human colonic microbiota are antagonistic *Bacteroides* (linked to a high intake of fat and protein) and *Prevotella* (linked to plant-rich diets with high levels of complex carbohydrates and fruit and vegetable intake), and interestingly, smoking causes an increase in both of them [35]. A commensal bacterium *Bacteroides*, in a myocarditis in mice model, triggers a cross-immune response against a bacterial protein and heart epitope, causing cardiomyopathy [82]. Moreover, *Prevotella* plays an important role in dysbiosis in pre- and hypertension patients [83], high lifetime cardiovascular disease risk [84], and cardiac valve calcification [85]. A positive correlation between *Catenibacterium* enrichment in current smokers and other unhealthy factors, such as the dietary level of animal fat, was revealed [86]. The study of Nolan-Kenney R. et al. showed a weaker relation in terms of the gut microbiome between former smokers and never smokers than comparing current smokers and never smokers, suggesting a beneficial effect of quitting smoking [36]. Class *Alphaproteobacteria*, presented in current smokers, consists, among others, of *Rickettsiales* that are capable of causing serious lesions of the mitral and aortic valves, leading to a need for valve replacement [87]. In a study involving 148 patients undergoing valve replacement, antibodies to *Rickettsia* spp. were detected in 12 cases [87].

Moreover, studies on newborns and infants suggest that both pre- and postnatal exposure to tobacco resulted in gut microbiome dysbiosis (enrichment in *Firmicutes*) that favors excessive body weight in children [88].

### 2.4. Physical Activity

Physical activity, leading to improved levels of cardiorespiratory fitness, is crucial in cardiovascular disease prevention [89] and it has been suggested to play a role in shaping the human gut microbiota [40,41,42,43,44,45,90,91]. Clarke S.F. et al. found that the gut microbiota of professional rugby players had greater alpha diversity and, compared to high-BMI controls, greater proportions of 48 taxa, while there was only a reduction in *Bacteroidetes* [40]. Further analysis showed that in the gut microbiome of elite athletes, compared to low-BMI controls, significantly higher proportions of 40 taxa were revealed with lower proportions of *Lactobacillaceae*, *Bacteroides*, and *Lactobacillus* [40]. Interestingly, both athletes and the low-BMI group demonstrated a higher proportion of the *Akkermansia* genus [41]. Additionally, identified microbiota representing 22 distinct phyla positively correlated with protein consumption and plasma creatine kinase [40]. In a follow-up study, the same group of researchers confirmed the microbial diversity among elite rugby athletes compared to nonnative, age-matched controls and again demonstrated higher levels of *Akkermansia* [42]. Additionally, active women (performing at least 3 h of exercise per week) compared with sedentary controls had increased levels of *Faecalibacterium prausnitzii*, *Roseburia hominis*, and *Akkermansia muciniphila* [41,43]. Another study on elderly women claims that aerobic exercise training may increase intestinal *Bacteroides* in association with improved cardiorespiratory fitness [44]. Two reviews from 2019 identified that the main phylum that respond to exercise is *Firmicutes* (including SCFA-producing genera from the *Firmicutes* phylum) [90,91].

In an interventional study on 26 subjects with abnormal glucose metabolism included in training modules, an increasing *Bacteroidetes* phylum and decreasing *Firmicutes*/*Bacteroidetes* ratio were revealed [45].

#### What Might This Mean for the Heart?

The most important change in gut microbiome composition that might be related to exercise is enrichment in the phylum *Firmicutes* [40,90,91] and the phylum *Verrucomicrobia*, which contains only a few described species [41,42,43]. One of them is *Akkermansia* that, as previously mentioned, is currently being considered as a next-generation therapeutic agent in obesity [41,92,93]. *Akkermansia *muciniphila**-derived extracellular vesicles seem to play a role in the regulation of gut permeability [94]. In a high-fat-diet-induced diabetic mice model, their administration enhanced tight junction function and occludin expression while reducing body weight gain and improving glucose tolerance [94]. Furthermore, not only extracellular vesicles but *Akkermansia* itself increased tight junction, such as occludin, occludin 4, zonula occludens 1–3, and the expression of Toll-like receptors 2 and 4 that participate in regulating the host immune system [95]. *Akkermansia muciniphila* also increases the number of goblet cells and mucus secretion, restoring the thickness of the intestinal mucus caused by a high-fat diet [96]. Its low abundance was also related to elevated body weight, blood cholesterol, and fasting blood glucose level [97]. A positive effect on health related to *Roseburia* enrichment due to exercise might be in relation to the fact that this bacterium in the study conducted on 4672 subjects from 6 different ethnic groups participating in the Healthy Life In an Urban Setting (HELIUS) was proven to be the best predictor from the machine learning model of blood pressure. A higher *Roseburia* abundance was related to a lower blood pressure [98]. *Roseburia* plays a role in maintaining gut health due to antimicrobial metabolites and immune defense, such as regulatory T-cell homeostasis, primarily through SCFA production (especially butyrate) [99]. These bacteria affect colonic motility and perform the maintenance of immunity and anti-inflammatory properties [99].

Another potentially positive aspect to the heart might be related to an increase in SCFA-producing genera from the *Firmicutes*, such as *Faecalibacterium prausnitzii* [100]. A gut microbiome dysbiosis expressed in the case of this bacteria as a depleted abundance was observed in chronic heart failure patients [101].

## 3. Association of the Microbiome with Nonmodifiable Risk Factors

### 3.1. Age

Emerging evidence from the literature suggests that aging is associated with the evolution of the human gut microbiome [46,47,48,49,50,102,103,104,105]. A comparative assessment from 2009 of the human fecal microbiota of infants, adults, and the elderly showed that the microbiota of infants were generally characterized by low levels of total bacteria abundance [46]. *Clostridium leptum* and *Clostridium coccoides* species were highly represented in the microbiota of infants, while elderly subjects exhibited high levels of *Escherichia coli* and *Bacteroidetes* [46]. Moreover, the *Firmicutes*/*Bacteroidetes* ratio changes from infants, to adults, to the elderly: 0.4, 10.9, and 0.6, respectively [46]. Another study, based on 367 healthy Japanese subjects between the ages of 0 and 104, showed changes in co-abundance groups (CAGs) related to age [47]. The dominance of *Bifidobacterium* (phylum *Actinobacteria*) was revealed for infants/children, whereas *Enterobacteriaceae* (phylum *Proteobacteria*) dominance was revealed for infants and the elderly [47]. Furthermore, *Lachnospiraceae* (phylum *Firmicutes*) was distinctive for adults and *Bacteroides* and *Eubacterium* and *Clostridiaceae*, *Megamonas*, and *Peptoniphilus* (phyla *Bacteroidetes*, *Firmicutes*) were distinctive for the elderly [97]. Moreover, sequential changes occurred in the relative abundance of *Bacteroides*, *Lachnospiraceae,* and *Bifidobacterium* in the gut microbiota during childhood and adolescence [47]. Additionally, Salazar N. et al. showed that the presence of the *Bacteroides*, *Bifidobacterium*, *Faecalibacterium*, and *Clostridium cluster XIVa* decreased with age up to the ages of 66–80, with differences reaching statistical significance for the latter group [50]. Interestingly, the abundance of some of these microorganisms changed again in the very old age group (>80 years), with these older individuals presenting significantly higher counts of *Akkermansia* and *Lactobacillus* than adults and the younger elderly [50].

Some authors have tried to identify the gut microbiome profiles responsible for longevity. In 2015, Wang F. et al. compared the gut microbiome of eight centenarians (100–108 years old) with eight younger elderly people (85–99 years old) and eight elderly people (80–92 years old), revealing a decrease in *Lactobacillus*, *Faecalibacterium*, *Parabacteroides*, *Butyricimonas*, *Coprococcus*, *Megamonas*, *Mitsuokella*, *Sutterella,* and *Akkermansia* and an increase in *Roseburia* and *Escherichia* [49]. In the study, *Ruminococcaceae*, *Clostridiaceae*, and *Lachnospiraceae* were characterized as age-related operational taxonomic units (OTUs)*,* and the former two were increased in centenarians [49]. Odamaki T. et al. showed a decrease in *Faecalibacterium*, *Roseburia*, *Coprococcus*, and *Blautia* and an increase in *Enterobacteriaceae* in the groups of analysis of 90- and 100-year-old subjects [47]. Interestingly, the bifidobacterial capacity to promote longevity by enhancement of bacterial polyamine biosynthesis has been shown in an animal model [103]. *Bifidobacterium breve* was detected in approximately 70% of children under 3 years old. *Bifidobacterium adolescentis* and *Bifidobacterium catenulatum* groups were predominant after weaning. *Bifidobacterium bifidum* was detected at almost all ages, from 0 to 104 years. The detection rate of *Bifidobacterium dentium* was higher in the elderly than in other age groups [48]. In another study, co-cultivating *Bifidobacterium* species resulted in an increase in the transcription of the gene required for exopolysaccharides biosynthesis [104]. This might influence the interaction between *Bifidobacterium* and the host, including the ability of commensal bacteria to remain immunologically silent and, in turn, provide pathogen protection [104].

Moreover, a decrease in stool microbial diversity is shown when we consider not chronological but biological age [105]. In one study, the subject frailty index (FI) and chronological age were assigned to low, middle, and high tertiles for OTU differential abundance analysis. Subjects with high frailty index scores, compared with those with a low frailty index, exhibited an increased abundance of the *Firmicutes* phylum (*Coprobacillus*, *Dialister)* and the *Saccharibacteria* phylum (*TM7 candidate-phylum*). A high frailty index was associated with a decrease in *Bacteroidetes* and *Proteobacteria* phyla (*Paraprevotella*, *Rikenellaceae*, and *Sutterella*, respectively). The middle frailty index, compared with a low frailty index, was associated with greater *TM7 candidate-phylum* abundance [104]. Furthermore, in the study, authors using the Sparse Inverse Covariance Estimation for Ecological Association and Statistical Inference and Weighted Gene Co-Expression Network Analysis identified modules of coabundant microbial genera related to biological or chronological age [105]. The positively coabundant *Eggerthella*, *Coprobacillus*, and *Ruminococcus* genera were significantly associated with biological age after correction for other confounders, for instance, body mass index, sex, and antibiotic usage in the past 6 months [105]. Therefore, this module might be considered a biological age indicator.

#### What Might This Mean for the Heart?

The data from the literature demonstrated that taxa enrichment in elderly people is potentially pathogenic [106,107]. The first of them is *Escherichia coli*, which could be a cause of endocarditis [106]. *Clostridium difficile* [107] was found on naive pathologically changed heart valves that were explanted [107].

Despite the fact that results from various studies differ in terms of *Megamonas* abundance in relation to advanced age [46,49,108], this taxon was depleted in heart failure patients [109] and in cardiac valve calcification patients [85]. Furthermore, according to the association with dyslipidemia, *Megamonas*, among others, was regarded as a potential pathogen for cardiovascular diseases [85].

Additionally, one of the OTUs related to age was *Ruminococcaceae* [49,51], the taxon connected to a healthy high-fiber diet [49] and enhanced short-chain fatty acid production [109,110,111]. However, this taxon was also found in the group with a vitamin D concentration < 50 nmol/L [112].

### 3.2. Gender

The differences in cardiovascular conditions between genders are related to genetic and hormonal differences between women and men [113]. Furthermore, this might be partially related to their distinct gut microbiome profiles [52,53,54,55,56]. Another important finding is the fact that data from the literature suggest that women may harbor a higher *Firmicutes*/*Bacteroidetes* ratio than men [52,53,54] and higher proportions of *Firmicutes* after adjusting for BMI [55]. Furthermore, among people with a BMI greater than 33, a significantly higher *Firmicutes*/*Bacteroidetes* ratio was seen in women compared to men, while the opposite value of the ratio was observed in those with a BMI less than 33 [55]. Other studies showed an increase in *Treponema* in women and increased *Eubacterium* and *Blautia* in men [56]. Furthermore, three species from *Clostridia*, one from *Bacteroidetes*, and two from *Proteobacteria* had a higher abundance in males than in females [53].

Moreover, an analysis conducted on 277 healthy Japanese subjects aged 20–89 years revealed increases in various genera related to age and depending on gender [51]. Increases in *Prevotella*, *Megamonas*, *Fusobacterium*, and *Megasphaera* were found in males, whereas *Bifidobacterium*, *Ruminococcus*, and *Akkermansia* were found in female subjects [51].

#### What Might This Mean for the Heart?

A higher *Firmicutes*/*Bacteroidetes* ratio, which was reported in women, is generally considered an indicator of gut dysbiosis. Its elevated value is reported in industrialized diets [56] and many cardiological diseases such as coronary artery disease [114,115] and myocarditis [116]. *Bifidobacterium* could be both beneficial and unfavorable to our health. Increased *Bifidobacterium* contributes to the generation of favorable microbial metabolites [117], attenuated cardiac injury from ischemia-reperfusion [118] and has been found in heart failure patients [108]. *Bifidobacterium* is often used as a probiotic and might act via modification of the gut microbiota, competitive adherence to the mucosa and epithelium, strengthening of the gut epithelial barrier, and modulation of the immune system to convey an advantage to the host [119]. Homeostasis with the host might be mediated by Toll-like receptors and nucleotide-binding oligomerization-domain-containing protein-like receptors, nuclear factor-ĸB, and mitogen-activated protein kinase [119].

Moreover, the depletion of *Blautia* genera was found in valve calcification [85]. *Prevotella* was connected to a diet rich in non-digestible carbohydrates, decreasing with elevated alcohol consumption [120], and is associated with pre- and hypertension [83].

## 4. *Firmicutes*/*Bacteroidetes* Ratio

**Figure 1:**
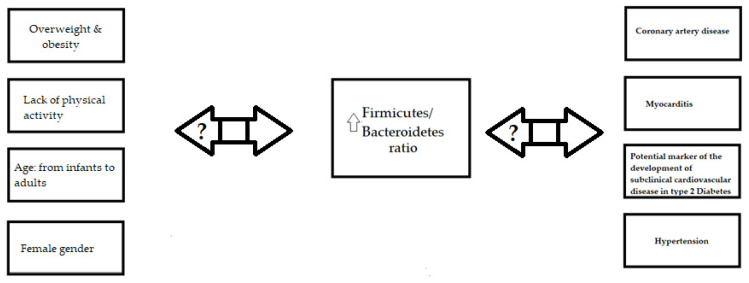
Firmicutes/Bacteroidetes ratio and cardiovascular risk factors and pathologies. Some cardiovascular risk factors influence Firmicutes/Bacteroidetes ratio, and Firmicutes/Bacteroidetes ratio might influence cardiovascular risk factors and cardiovascular pathologies. It is questionable whether a higher Firmicutes/Bacteroidetes ratio is a cause or a result of cardiovascular pathologies and if cardiovascular pathologies could affect Firmicutes/Bacteroidetes ratios.

In the literature, different gut microbiota were related to risk factors, particularly for cardiovascular diseases; however, one of the most common results is a change in the *Firmicutes*/*Bacteroidetes* ratio [19,83,84,85,86,87,114,115,116,121,122,123]. A higher value was found to be related to abnormal body weight, lack of physical activity, and female gender (Figure 1) [19,52,53,54,90,112,113]. Furthermore, the *Firmicutes*/*Bacteroidetes* ratio changes with age (Figure 1) [46].

## 5. Short-Chain Fatty Acids and Cardiovascular Risk Factors

Short-chain fatty acids (SCFAs) are products of the fermentation of non-digestible carbohydrates by the gut microbiota in the human digestive tract [124]. Among them, propionate and butyrate seem to play the most significant health-promoting roles [125]. SCFAs are absorbed by diffusion or via GPR41 and GPR43 in the intestine [126]. A normal colonic epithelium derives 60–70% of its energy supply from SCFA-butyrate [127]. Propionate is transported to the liver and used as a precursor for gluconeogenesis, liponeogenesis, and protein synthesis [128], while acetate can be used as a substrate for lipogenesis and gluconeogenesis [125,129]. SCFAs may also target the liver to reduce hepatic glucose production and lipid accumulation via AMPK activation [126]. SCFAs can directly signal to adipose tissue via GPR43/GPR41 and enhance adipogenesis and uncoupling protein-1 production while restraining lipolysis and inflammation [126]. Furthermore, SCFA might target the brain via GPR41 and also stimulate the production of satiety hormones (glucagon-like peptide 1 and protein YY) production [126]. In the study of Frost G. et al., acetate was shown to accumulate in the hypothalamus and induce acetyl-coenzyme carboxylase activation A, causing a change in the expression profile of regulatory neuropeptides and leading to appetite suppression [130].

**Figure 2:**
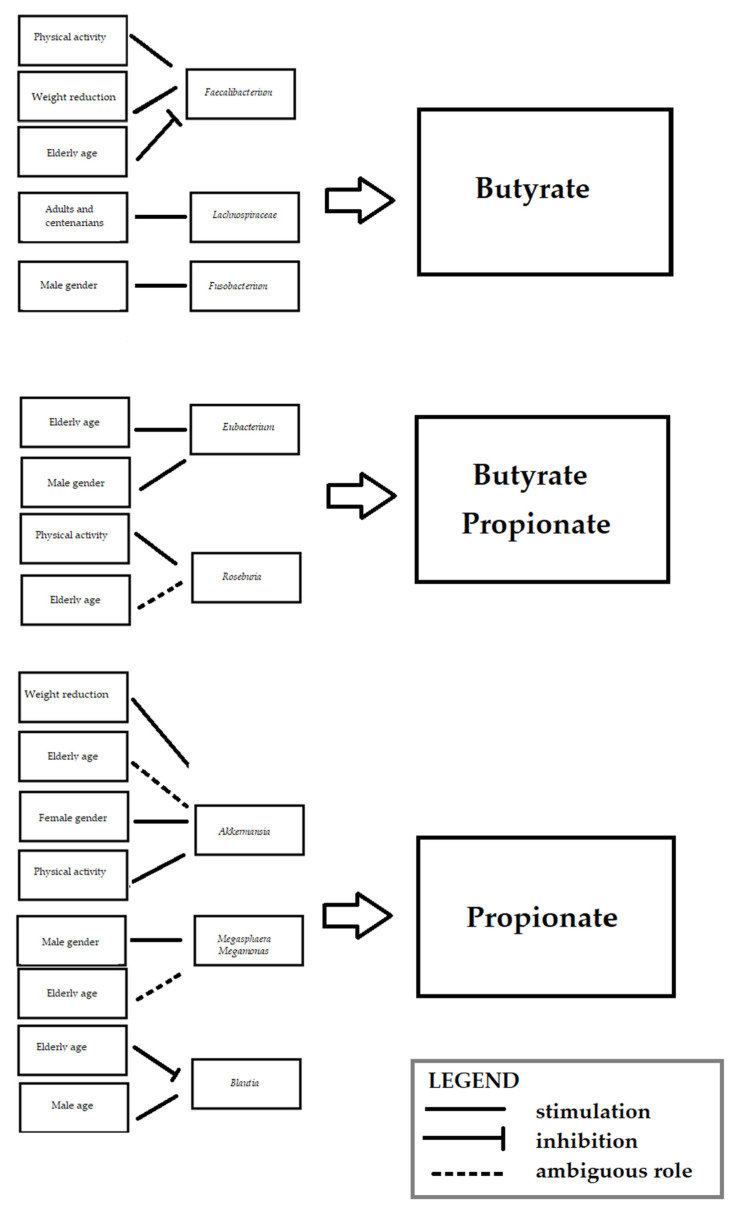
Short-chain fatty acid production potential of listed bacteria and their association with particular cardiovascular risk factors.

In this review, we underlined the fact that some bacteria related to particular risk factors are involved in SCFA production (Figure 2).

## 6. Summary

In this review, we discussed why particular microbes occur in individuals and how they might influence the host’s cardiovascular system in health and disease, especially in the context of modifiable and nonmodifiable cardiovascular risk factors.

We identified many bacteria that are connected to risk factors and responsible for specific diseases; however, the main finding is that the most important factor is the whole gut’s microbiome composition and the shifts in it. It is commonly suggested that the *Firmicutes*/*Bacteroidetes* ratio is an indicator for gut dysbiosis, and one study found its increase in overweight and obesity and its changes due to aging and gender. Furthermore, we underlined the fact that some bacteria related to cardiovascular risk factors are involved in SCFA production. Despite the enormous knowledge about cardiovascular risk factors and the gut microbiome, some influences, interdependencies, and relationships are still not fully elucidated. Therefore, studies based on a larger population with advanced, novel “omics” methods should be conducted.

## 7. Future Area of Research

It is essential to be aware that the gut microbiome’s composition is susceptible to many environmental factors other than those described in this manuscript, for instance, medication, diet [131], and environmental factors (temperature, habits, pollution). Furthermore, it is still not fully elucidated whether changes in the microbiome are a cause or an effect of cardiovascular diseases. To answer this question, prospective cohort studies on large populations examining gut microbiome composition before and after disease onset are necessary. Another important aspect is the incorporation of microbiome analyses in randomized controlled trials. In the future, particular benefits could be obtained from interventional studies on cardiovascular risk factor modification by microbiota transplantation.

Due to the fact that many mechanisms concerning gut microbiota described in this manuscript have only been tested in animal models, the significance of these phenomena should be documented in human models as well, with an emphasis on the distinctiveness of the gut microbiota in individual species.

## Figures and Tables

**Table 1:** Association of microbiome with modifiable risk factors.

Risk Factor		Increase In	Decrease In
Overweight and obesity	Observational study:	- *Firmicutes*/*Bacteroidetes* ratio [19]	- Gut microbiome diversity and richness [20]
	Interventional study:	- Phylum *Bacteroidetes*: transmitted from lean mice [21] - Phylum *Actinobacteria*: genus *Bifidobacterium* in probiotics group [22] - After weight reduction: total bacterial abundance, Lactobacilli, *Clostridium cluster* IV, *Faecalibacterium prausnitzii*, *Archaea*, *Akkermansia* [23]	- Phylum *Bacteroidetes*: *Bacteroides vulgates* in probiotics group [22] - After weight reduction: *Firmicutes*/*Bacteroidetes* ratio *Clostridium cluster XIVa* [23]
Cholesterol-rich lipoprotein metabolism	Observational study:	- Phylum *Firmicutes*: family *Clostridiaceae*/*Lachnospiraceae* with LDL; genus *Coprococcus* with triglyceride [24] - Phylum *Actinobacteria*: genus Collinsella species Stercoris with triglycerides [24] - Phylum *Proteobacteria*: family *Pasteurellaceae* with triglycerides [24] - Phylum *Bacteroidetes*: *Bacteroides dorei* lower serum total cholesterol [25] - Phylum *Firmicutes*: *Eubacterium coprostanoligenes* lower serum total cholesterol [26], *Lactobacillus* sp. lower serum total cholesterol [27], *Lactococcus* lower total serum cholesterol, and LDL [28,29,30], cause reduced absorption of sterols - Phylum *Actinobacteria*: *Bifidobacterium* sp. lower serum total cholesterol [25,31]	- Richness and diversity [32]
	Interventional study:	- Phylum *Proteobacteria*: *Desulfovibrionaceae* in diet enriched with bile acid and lard and palm oil in mice model, *Ruminococcaceae* diet enriched with lard [33]	- Phylum *Firmicutes*: *Erysipelotrichaceae* in diet enriched with bile acid and lard and palm oil in mice model, *Lachnospiraceae* in diet enriched with lard [33] - Phylum *Firmicutes*: *Lactococcus* lower total serum cholesterol and LDL [28,29,30], cause reduced sterols absorption [31,34]; *Enterococcus faecium* lower total serum cholesterol and LDL [28,29,30]
Tobacco smoking	Observational study:	- Phylum *Proteobacteria*: [35]; *Alphaproteobacteria* [36] - Phylum *Bacteroidetes*: genera *Bacteroides* and *Prevotella* [35,37]; *Prevotella* in electronic cigarettes [38]; genus *Bacteroides* in 6-month-old infants [39] - Phylum *Firmicutes*: genus *Clostridium* [35]; *Erysipelotrichai*-to-*Catenibacterium* [36]; *Ruminococcus* in neonates [39]; genus *Staphylococcus* in 6-month-old infants [39] - Increased gut bacterial richness, particularly *Firmicutes* in 3-month-old infants [39] - Phylum *Verrucomicrobia*: *Akkermansia* in neonates [39]	- Gut microbiome diversity [35] - Phylum *Actinobacteria*: genus *Bifidobacteria* [35] - Phylum *Firmicutes*: genus *Lactococcus* [35] - Phylum *Proteobacteria* [37] - Phylum *Bacteroidetes*: *Bacteroides* in electronic cigarettes [38]
Physical activity	Observational study:	- Gut microbiome diversity [40] - 48 taxa vs. high-BMI controls [40] - 40 taxa vs. low-BMI controls [40] - Phylum *Verrucomicrobia*: *Akkermansia* [41,42,43] - Phylum *Firmicutes*: *Roseburia hominis*, *Faecalibacterium prausnitzii* [43] - Phylum *Bacteroidetes*: genus *Bacteroides* [44]	- Phylum *Bacteroidetes* vs. high-BMI controls [40] - Phylum *Bacteroidetes*: *Bacteroides* vs. low-BMI controls [40] - Phylum *Firmicutes*: genus *Lactobacillus* vs. low-BMI controls [40]
	Interventional study:	- *Bacteroidetes* [45]	- *Firmicutes*/*Bacteroidetes* ratio [45]

**Table 2:** Association of microbiome with non-modifiable risk factors.

Risk Factor		Increase In	Decrease In
Age	Observational study:	Neonates—children: - Phylum *Firmicutes*: *Clostridium* leptum and *Clostridium coccoides* for infants [46] - Phylum *Proteobacteria*: *Enterobacteriaceae*: for infants and the elderly [47] - Phylum *Actinobacteria*: *Bifidobacterium* for infant/child [47]; *Bifidobacterium breve* in children [48] Adult: - Phylum *Firmicutes*: *Bifidobacterium adolescentis* and *Bifidobacterium catenulatum* after weaning [48]; *Lachnospiraceae* for adults [47] Elderly: - Phylum *Proteobacteria*: species *Escherichia coli* in the elderly [46]; *Roseburia* in the centenarians [49]; *Enterobacteriaceae* in group 90–100 years old [47]; *Enterobacteriaceae* for infants and the elderly [47] - Phylum *Bacteroidetes*: *Bacteroides* [47]; in the elderly [46] - Phylum *Firmicutes: Clostridiaceae*, *Eubacterium*, *Megamonas* and *Peptoniphilus* for the elderly [47]; *Lactobacillus* group in the elderly (>80 years old) [50]; *Clostridiaceae*, *Lachnospiraceae* in the centenarians [49] - Phylum *Verrucomicrobia*: *Akkermansia* in the elderly (>80 years old) [50] - Phylum *Actinobacteria*: *Bifidobacterium dentium* in the elderly [48]	Neonates—children: - Gut microbiome diversity in infants [46] - *Firmicutes*/*Bacteroidetes* ratio in infants and the elderly [47] Elderly: - Phylum *Bacteroidetes*: *Bacteroides* in elderly [50]; *Parabacteroides*, *Butyricimonas* in the centenarians [50] - Phylum *Actinobacteria*: *Bifidobacterium* in the elderly [50] - Phylum *Firmicutes*: *Clostridium cluster XIVa*, *Faecalibacterium* in the elderly [50]; *Faecalibacterium*, *Coprococcus*, *Lactobacillus*, *Megamonas*, *Mitsuokella* in the centenarians [49]; *Faecalibacterium* in group 90–100 years old [47]; *Lactobacillus* in the elderly (>80 years old) [50]; *Roseburia*, *Coprococcus*, *Blautia* in group 90–100 years old [47] - Phylum *Proteobacteria*: *Sutterella* in the centenarians [49] - Phylum *Verrucomicrobia*: *Akkermansia* in the centenarians [49]
Gender	Observational study:	Female: - Phylum *Verrucomicrobia*: *Akkermansia* [51] - Phylum *Actinobacteria*: *Bifidobacterium* [51] - Phylum *Firmicutes*: *Ruminococcus* [51] - *Firmicutes*/*Bacteroidetes* ratio [52,53,54] - *Firmicutes*/*Bacteroidetes* ratio in females with a BMI greater than 33 kg/m^2^ [55] - Phylum *Firmicutes* adjusting for BMI [55] - Phylum *Spirochaetes*: *Treponema* [56] Male: - Phylum *Fusobacteria*: *Fusobacterium* [51] - Phylum *Proteobacteria*: two species [53] - Phylum *Bacteroidetes*: one species [53], *Prevotella* [51] - Phylum *Firmicutes*: *Eubacterium* and *Blautia* [56]; three species from *Clostridia* [53]; *Megamonas* and *Megasphaera* [51]	Female: - *Firmicutes*/*Bacteroidetes* ratio in females with a BMI less than 33 kg/m^2^ [55]

## Data Availability

Not applicable.
